# The Mystery of the Royal Touch

**Published:** 1910-08-13

**Authors:** 


### SPECIAL ARTICLES.

### THE MYSTERY OF THE ROYAL TOUCH.

The practice of healing by touch can be traced back to a very early period. It was a survival of a rite performed by the priest-physicians of ancient Egypt and Babylonia, and later Pyrrhus and Vespasian cured disease by touch. It probably took its rise in the ancient belief that certain individuals were born with superior powers to those possessed by their fellow-creatures. Monarchs were exalted above others on account of the supposed distinctive excellence imparted to them at their birth by the ruling signs and planets. It was but natural therefore that their subjects believed them capable of imparting this influence by a glance of the eye or a touch of the hand. To touch even their robes was sometimes thought sufficient to receive a share of their special virtues. Thus arose the belief in the healing power imparted by a touch of the royal hand of the Lord's anointed, a superstition deeply rooted in the human mind.

### How the Confessor Touched.

The royal power of healing by touch is said by the early chroniclers to have been a prerogative peculiar to the sovereigns of England. The practice in this country appears to have originated with Edward the Confessor, who reigned A.D. 1041-1066, from whom the hereditary right is said to have descended to all his successors to the English throne. It is first recorded by William of Malmesbury, who quaintly relates the story of "a young woman who had a husband about the same age, but having no child, got an ill state of health by an overflowing of humours in her neck, which broke out in great nobbs. That she was commanded in a dream to apply to the King to wash it. To Court she goes and the King being at his devotions all alone, dip'd his fingers in water and dabbel'd the woman's neck and he had no sooner taken away his hand, but she found herself better, the loathesome scabb dissolved, but the lips of the ulcers remaining wide and open she remained at Court till she was well, which was in less than a week's time, the ulcers being so well closed, the skin so fair that nothing of her former disease could be discerned, and in a year's time she was brought to bed of twins!"

King Edward also seems to have exercised his healing power in other directions, and a story is recorded of his meeting with "a cripple much diseased, whom he carried on his back to St. Peter's Church in Westminster, whereupon he was immediately cured of all his maladies."

In the "Computus Hospitii" of Edward I. it is recorded several times that a small sum of money (gold) was given by the King to those he healed. Edward the Confessor does not seem to have limited his power of healing to one complaint, as was the case with his successors, who claimed the right only to heal scrofula or struma. This disease was commonly known as King's Evil, and was so called, says an old writer, "because the King's touch could cure it." Shakespeare alludes to the Confessor's healing touch in *Macbeth* (Act IV., scene 3).

### The Whole Art of Touching.

Certain kings of France, however, claimed the right of healing by touch, including Philip I., Charles X., and Henry IV. Louis XIV. also practised it extensively, and is said to have touched 1,600 persons on Easter Sunday in the year 1686. While touching the afflicted he is said to have used the words, "The King touches you. May God heal you," and to each Frenchman was given 15 sous and every foreigner 30 sous. Laurentius, physician to Henry I. of France, asserts that this gift of healing belonged only to the kings of France, and that it was first practised by Clovis in the year 481, who discovered his power accidentally when he caused the disappearance of a painful chronic swelling from the neck of his follower Lanicetus by laying his hand upon it. The English chroniclers maintain, however, that the kings of France only derived their power from their alliance to the English Royal line, and this matter in ancient times was the subject of many a bitter controversy. The French kings exercised their virtue longer than our English sovereigns, and kept up the practice until the year 1776.

After the time of Edward I. no mention is made by the chroniclers of the practice of healing by touch until the reign of Henry II., when Peter de Blois, who was chaplain to that monarch, records that "his master touched and cured scrofula." Gilbertus Anglicus, who is said to have lived in the time of King John, in his work called "Compendium Medicinæ" refers to the practice as an ancient one, and says "the disease was called King's evil because the Kings cured it."

The next allusion is that made by John of Gaddesden, who is said to have been the first English physician employed at Court. Writing on scrofula and enumerating the various methods of treatment for the disease about 1320, he recommends, in case of failure, that the patient should repair to the King to be touched.

The gift of a coin with supposed amuletic virtue doubtless aided the continuance of the practice, and many probably had more faith in the touch-piece than in the sacred hand of its giver.

John Browne, surgeon to Charles II., states, "the giving of the gold coin or touch-piece was a token of good will from his Majesty and was not supposed to be essential to the cure."

For all that, many of the sufferers appear to have regarded the piece as very essential to the cure, and if they happened to lose or probably having sold it, they often applied for another token of "His

Majesty's good will." The piece was most likely regarded as an amulet to be worn to prevent the return of the disease, as other coins were worn in the Middle Ages as charms to ward off epilepsy and certain diseases. Coins called the money of St. Helena were esteemed especially efficacious in epilepsy, and mention is made of them in the wardrobe accounts of Henry III.

In Henry VII.'s time a special form of service was first used for the performance of the curious ceremony of healing by touch.

Beckett declares that "this service was adapted from an ancient exorcism called 'Thesaurus Exorciomorum atque conjurationium Terribilium, which was used for the dispossessing of Evil Spirits. The Gospels ordered to be read and many of the phrases are the same in each service."

### Good Queen Bess as a Healer.

Although Elizabeth was said to have been averse to the practice, the maiden queen tested her healing virtue when she came to the throne, and is said to have touched an immense number of people. Richard Wiseman exults "that the gift was not taken away upon our departure from the Church of Rome," for it was regarded as a proof of rightful sovereignty. According to Richard Smith, Queen Elizabeth made the sign of the cross with her finger on the scrofulous swellings, a practice which was not followed by her successors until James II. revived it. From a speech she is said to have made on one of her journeys through Gloucestershire, Elizabeth showed her dislike to practise the healing, for on being importuned by many persons suffering from the disease, she exclaimed, "God only could relieve them from their complaints."

William Clowes, who was her Majesty's surgeon, in his book entitled "A Right Frutefull and Approved Treatise for the Artificiall cure of that Malady called, in Latin Struma, and in English the Evill, cured by Kingis and Queenes of England," describes the disease as "one repugnant to nature, which grievous malady is known to be miraculously cured and healed by the sacred hands of the Queene's most royall majesty even by divine inspiration and wonderfull worke and power of God above man's skill, arte and expectation."

The Stuart kings appear to have had a special liking for exhibiting the virtue of the royal touch. Among those who were brought to James I. was the son of the Turkish Ambassador, who, it is said, "was troubled with a diseased throat and was stroked by King James and said to have been cured."

### Methods of Healing.

The method of applying the hand appears to have been varied by certain sovereigns. Before Queen Elizabeth's time we have only the mention of the sores being touched. Elizabeth contented herself by making a sign of the cross in the afflicted part, but her successors, James I., Charles I., and Charles II., are said to have performed the rite by "stroking" the swellings. Charles I. is said by a contemporary writer "to have excelled all his predecessors in the divine gift, for it is manifest beyond all contradiction that he not only cured by his sacred touch both with and without gold, but likewise perfectly effected the same cure by his prayer and benediction only." He certainly "stroked" a large number of people, and Wiseman relates that when the King's fortunes had fallen so low that he could not give gold touch-pieces, he was the first to have special coins struck for the purpose, of silver and even copper. The copper touch-piece is now rare, and bears on one side a hand with the words "He touched."

### In the Time of the Merry Monarch.

After the Restoration multitudes of people flocked to receive the benefits of the royal touch, and in his performance of the ceremony Charles II. numerically surpassed all his predecessors. This may perhaps be accounted for owing to the suspension of the rite during the time of the Commonwealth, although John Browne, the author of a curious little work entitled "Adenochoradelogia," states that "the method had been tried by 'the late usurper Cromwell,' but without success."

Charles II. "stroked" several thousands of people every year, and in 1682 he performed the rite 8,500 times. Parish registers were kept of such cases as had received certificates and were touched for the King's Evil. One of these, at Camberwell, is dated 1684, and others are at Stanton St. John near Oxford, Wadhurst in Sussex, and King's Langley in Hertfordshire.

From the public register kept by Thomas Haynes, Serjeant of his Majesty's Chapel Royal from 1660 to 1664, 23,601 were touched, and again from 1667 to 1684 the number of people touched by Charles II. reached 68,506.

So great indeed was the increase in the number of applicants that it was soon found necessary to place some restrictions upon them. "That none may approach his Royal presence but such as are really troubled with the Evil," says Browne, "several officers were appointed for this great ceremony. Among the first of which are his Majesty's Chirurgeons-in-waiting, who are to take in certificates and deliver out tickets in order to a healing." Patients were bound, when applying for a ticket, to bring with them a certificate signed and sealed by their ministers and churchwardens, declaring they had never before been "touched" by the King.

### Faith-Healing.

That faith played a prominent part in the healing is evidenced from the statement by Browne, that "the healing held on Good Friday do carry a strong faith with some people, who, unless they can be 'touched' by the King that time, their belief is so weak and tender that they do presume and suppose any other time in the year is not so fitting." Browne gives a number of letters from persons who profess to have been cured by the "touch," which read something like the testimonials in an advertisement of a modern patent medicine. The operation was performed with much solemnity, and the ceremony usually took place on a Sunday. In winter it was always held at Whitehall, but in summer it was sometimes held at Windsor.

According to the advocates of the remedy, none failed to receive benefit unless their little faith starved their merits. Some are said to have been cured immediately they were touched, while others did not get rid of their swellings until "touched" a second time. Cases are related of persons who had been quite blind for several weeks, and were even obliged to be led to Whitehall, yet recovered their sight immediately on being "touched"; others who could not walk were brought on beds, and are said to have received immediate relief.

In the *Parliamentary Journal* for July 2-9, 1660, is the following allusion:—

"The Kingdom having been for a long time troubled with the Evil by reason of his Majesty's absence, great numbers have lately flocked for cure. His sacred Majesty on Monday last touched 250 in the Banquetting House, among whom, when his Majesty was delivering gold, one shuffled himself in out of an hope of profit which had not been stroked, but his Majesty quickly discovered him saying, 'This man hath not yet been touched.' His Majesty hath for the future appointed every Friday for the cure, at which 800 and no more are to be presented to him, who are first to repair to Mr. Knight, the King's Surgeon, being at the 'Cross Guns' in Russell Street, Covent Garden, over against the Rose Tavern, for their tickets."

### Some References.

Pepys, in his "Diary," makes the following reference to the ceremony, of which he was a witness:—

"April 13, 1661: I went to the banquet house and there saw the King heale, the first time that ever I saw him do it; which he did with great gravity, and it seemed to me an ugly office and a simple one." Evelyn gives a more detailed account, which is dated July 6, 1660.

The eagerness to obtain certificates from the chirurgeons was so great that Evelyn says, March 28, 1684: "There was so greate a concourse of people with their children to be touch'd for the evil that 6 or 7 was crush'd to death by pressing at the chirurgeon's doore for tickets." Charles II. was importuned by the afflicted even when out walking, and according to Ashmole, a man named Evans, who was in such a loathsome condition that no one could be found willing to recommend him for a certificate, placed himself in St. James's Park, where he knew the King walked. Upon his approach he fell on his knees, exclaiming, 'God bless your Majesty!' whereupon the King gave him his hand to kiss, upon which Evans availed himself of the opportunity to apply it to his dreadfully ulcerated nose, which from that time improved and ultimately recovered."

It is a significant fact that according to the bills of mortality of the period more people are said to have died from scrofula during the reign of Charles II. than at any time previous.

John Bird, a firm believer in the efficacy of the Royal touch, was of the opinion that the healing power was not confined to one disease, but that rickets could be cured as well as scrofula. In support of his belief he wrote a book.

James II. practised the healing like his predecessors, and according to Bishop Cartwright, on August 28, 1687, he touched 350 persons at one sitting. On another occasion he is said to have performed on some 800 sufferers in the choir of Chester Cathedral.

### Later Opinions.

William III. is said to have considered the rite "a silly superstition," and could only be induced to go through the ceremony once, when he said to the patients: "God give you better health and more sense!" But so firm was this belief rooted in the minds of the people it survived King William's ridicule, and was revived by Queen Anne, who performed the rite both in Oxford and in London. On March 30, 1714, she "touched" 200 persons, among whom was Samuel Johnson, who had been brought from Lichfield for the purpose by his mother.

So late as February 28, 1712, a little more than two years before her death, the following proclamation appeared in the *Gazette*:—

"It being Her Majesty's royal intention to touch for the Evil on Wednesday, the 19th of March next, and so to continue weekly during Lent, it is Her Majesty's command that tickets be delivered the day before at the office in Whitehall; and that all persons shall bring a certificate signed by the Minister and Churchwardens of their respective parishes that they have never received the royal touch."

### The Last Royal Healer.

Although Queen Anne was the last sovereign of England to practise the royal touch, both Prince Charles Edward, "the young Pretender," and also the so-called James VIII. claimed to have inherited the power, and had special silver "touch" pieces struck for the purpose. Thomas Carte, the historian, states that to his certain knowledge the Pretender had cured the scrofula, and argued therefrom that the healing virtue was transmitted in the true line of succession independently of any unctions.

There can be no doubt that some of the sufferers who flocked to "the healing" received a certain benefit from the treatment, but it should be also remembered that the distinction between strumous and other swellings in those days were often unrecognised even by physicians of repute. Many who applied to be touched were probably only suffering from swollen glands or simple boils which would in a short time have disappeared with ordinary treatment. In other cases faith or the influence of the imagination played an important part. "Imagination," says Lord Bacon, "is next akin to miracle—a working faith." And so the beneficial effect that in some cases is said sometimes to have followed the royal touch was no doubt in a great measure due to the strong feeling of hope and certainty of cure engendered in the mind.

An echo of this belief exists to-day, and is manifested in the followers of Mrs. Eddy and the cult of faith-healers.

